# A Survey on the Burden and Gaps in the Management of Atopic Dermatitis: Perspectives From Indian Dermatologists

**DOI:** 10.7759/cureus.91567

**Published:** 2025-09-03

**Authors:** Komal Gaur, Bhushan Kothawade, Sreenath Hariharan, Charles V Adhav

**Affiliations:** 1 Dermatology, Pfizer Ltd., Mumbai, IND; 2 Pharmacology, Pfizer Ltd., Mumbai, IND

**Keywords:** allergy and immunology, atopic, dermatitis, dermatologists, dermatology, disease management, surveys and questionnaires

## Abstract

Background: The burden of immuno-inflammatory diseases, such as atopic dermatitis (AD), is increasing, and data on their epidemiology and management from Indian settings are scarce. This study assesses the perspective of Indian dermatologists on AD burden, management, and unmet needs.

Methodology: This was an online questionnaire-based survey that captured the perspectives of 150 Indian dermatologists on AD prevalence, signs and symptoms, management, and treatment adherence.

Results: Most dermatologists reported that 5-10% of their patients had AD, with pediatric-onset comprising >60% of cases. The reported severity was mild in 41-60% of cases, moderate in 21-40%, and severe in <20% of cases. As per participating dermatologists, >60% and 21-40% of patients had a family history of atopy and AD, respectively. The most commonly reported signs and symptoms included itch, dryness, and sleep disturbances, with itch being proportional to disease severity in more than 60% of patients. Most mild AD cases experienced one to five flare-ups per year, as reported by 84.67% of dermatologists. Moderate cases had one to five flare-ups annually, as per 64.67% of the dermatologists. Severe cases were reported to experience six to 10 flares/year. Flare triggers included hot/humid conditions, stress, and a history of atopy. Topical corticosteroids and calcineurin inhibitors were the preferred first-line therapies for mild/moderate AD, whereas severe cases often required systemic conventional therapies and off-label drug usage with targeted therapies. Topical calcineurin inhibitors and moisturizers were the most commonly preferred for AD maintenance. Most dermatologists reported nonadherence to treatment due to loss of follow-up in <25% of patients. Notably, 78% believed that newer steroid-sparing options would improve adherence. Regional variations in onset and treatment preferences were also observed.

Conclusion: Significant gaps exist in the management of AD in India. Targeted therapies and newer steroid-sparing options could enhance patient outcomes. Challenges encountered included delayed diagnosis, preference for alternative medicine, and inappropriate treatment. Nonadherence to treatment was influenced by steroid phobia, lack of awareness, and cost of therapy. The findings should be interpreted with caution due to limitations, including recall bias and the lack of patient-level data.

## Introduction

Atopic dermatitis (AD) is an immuno-inflammatory skin condition. According to the Global Burden of Disease study (1990-2017), it has the highest burden among dermatological disorders based on disability-adjusted life-years (DALYs) [1]. AD presents with a complex array of signs and symptoms, including persistent pruritus, sleep disturbances, and skin inflammation, which not only affect the physical health of patients but also significantly impair their social, emotional, and mental well-being. This often leads to reduced performance at work or school, social withdrawal, and considerable emotional stress for patients and their families, contributing to a diminished quality of life [1,2].

AD primarily develops during childhood, with most cases presenting within the first few years of life. However, cases of adult-onset AD are not uncommon and tend to be more severe and persistent compared with cases of childhood-onset AD [2,3]. Familial predisposition to AD has also been documented, with studies indicating a genetic component that increases the likelihood of disease development, particularly when one or both parents have a history of atopic conditions [4].

In addition to the physical and psychological aspects, managing AD presents significant challenges for both patients and healthcare providers. Patients with AD face considerable difficulties in managing the condition, including adherence to treatment regimens, managing flare-ups, and coping with the chronicity of the disease. Poor awareness and misconceptions about AD further exacerbate these challenges, often leading to delayed diagnosis and suboptimal treatment outcomes [5,6]. Moreover, patients often seek alternative treatments, complicating the management of AD even further [6].

The prevalence of AD in India is increasing [7]. Despite this rising prevalence, there is a lack of comprehensive epidemiological data on the condition in the Indian population. Moreover, regional variations in treatment practices and patient presentations may further complicate disease management. Therefore, such data are critical for understanding the actual burden of the disease and guiding healthcare systems in developing effective strategies for disease management [1]. Given the complexities, it is essential to understand the perspectives of healthcare professionals, particularly dermatologists, who are at the forefront of diagnosing and managing AD. Their insights can help identify gaps in the current treatment approaches and highlight areas where more focused interventions may be needed. However, there is limited data on healthcare professionals' perspectives regarding AD, its management, and the specific challenges they encounter [8].

This study aims to assess the perspectives of healthcare professionals across India on the burden, clinical presentation, and management of AD. Additionally, the study seeks to evaluate regional variations in the management of AD, as these can offer valuable insights into tailoring or standardizing treatment strategies to meet the specific needs of patients in different parts of the country [9].

## Materials and methods

Study design

This study was conducted as a questionnaire-based cross-sectional survey aimed at capturing the perspectives of Indian dermatologists regarding the burden, clinical presentation, and management of AD. The primary objective was to assess the prevalence, severity, signs, and symptoms of AD, management strategies employed by dermatologists in India, as well as treatment adherence and unmet needs in managing AD. This study adhered to the Checklist for Reporting of Survey Studies (CROSS) for designing and reporting the survey [10].

Survey development and validation

The internal Pfizer medical team developed the survey. The questionnaire underwent internal content validation for clarity, comprehensiveness, and clinical relevance. It comprised a structured set of questions designed to gather detailed information on various aspects of AD management, focusing on the following areas (Appendices): (a) Prevalence of AD in clinical practice: The frequency of AD cases seen by dermatologists is assessed based on the survey period, physician experience, and point prevalence observed during clinical practice in the month of the survey, (b) Age of onset: Differentiating AD cases based on pediatric, adolescent, and adult onset, (c) Severity distribution: Categorizing AD into mild, moderate, and severe cases. The severity was assessed based on the clinical experience of the investigating physician, (d) Family history: Documenting any history of atopy or AD in the patients' families, (e) Most concerning signs and symptoms: Identifying the signs and symptoms that most commonly affect patients' quality of life, (f) Correlation of itch Wwth disease severity: Understanding how the severity of itch relates to the overall disease burden, (g) Frequency of flares: Estimating the annual flare-up rates based on AD severity, (h) Triggers for AD flares: Assessing common factors (e.g., environmental, dietary, etc.) contributing to disease exacerbations, (i) Referral patterns: Documenting referrals from other medical specialties for AD management, (j) Challenges in managing patients with AD: Highlighting key difficulties encountered in AD treatment, (k) Preferred first-line and maintenance therapies: Identifying treatment preferences across various AD severity levels, (l) Treatment adherence issues: Examining factors contributing to patient nonadherence.

Survey administrations and procedures

The survey was distributed online using Google Forms. Pfizer’s field medical team contacted dermatologists across India through emails and other digital platforms to encourage participation. The dermatologists, all certified by the Medical Council of India (now the National Medical Commission) or respective State Medical Councils, were provided with a link to the survey and were invited to complete it at their convenience. Participation was voluntary, and responses were anonymized to maintain confidentiality. To capture diverse clinical perspectives, dermatologists from various geographical locations, representing both urban and rural areas, were encouraged to respond to the survey. To ensure the accuracy of survey data, measures were taken to prevent multiple participation by the same respondent. These included providing a unique, one-time-use survey link and requiring the participants to have unique email addresses. A manual review of responses was also conducted to eliminate duplicates.

Healthcare professionals were informed about the survey objectives prior to rollout, encouraging them to provide broad assumptions or averages derived from their overall clinical experience rather than recalling isolated cases, in order to mitigate recall bias. Nonetheless, this remains a limitation of the study.

Respondents

A total of 150 dermatologists from various parts of India participated in the survey. The selection used convenience sampling to include dermatologists based on their availability. Efforts were made to ensure representation from all four geographical zones of India. The inclusion criteria were limited to only those who were licensed and currently practicing in India, actively managing patients with AD in their practice, and willing to provide informed consent. The exclusion criteria included non-dermatology specialists or healthcare professionals without direct involvement in AD management and those who did not provide consent. All respondents were practicing dermatologists, ensuring the data collected was from professionals with relevant clinical experience in managing AD. The study sampled practicing dermatologists across India through professional networks and electronic communication platforms such as email and social media. This approach aimed to capture a diverse representation of dermatologists from different geographical regions (North, South, East, and West) and practice settings (urban and rural). Out of 220 invitations sent, 150 completed responses were received, resulting in a response rate of 68.18%, ensuring a broad and representative sample of dermatologists from across the country.

Data analysis

The data from the completed surveys were exported to Microsoft Excel for cleaning and analysis. A thorough data cleaning was performed to eliminate incomplete or duplicate entries. Frequencies and percentages were calculated for categorical variables. Ranking questions were summarized by the percentage of dermatologists selecting each option for Rank 1, Rank 2, and Rank 3. Responses were stratified by geographical zone (North, South, East, West) to assess regional variations in onset, management practices, and adherence challenges. Missing responses for individual questions were excluded from denominators when calculating percentages; no imputation was performed. All analyses were descriptive in nature, given the exploratory intent of the study.

## Results

Epidemiology and demographics

**Figure 1:**
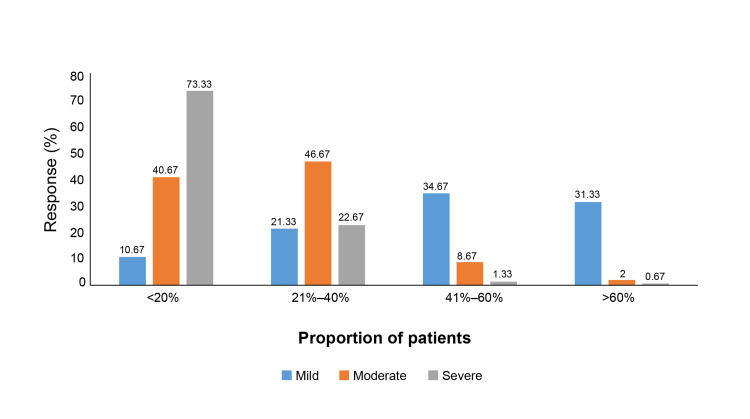
Severity proportions of mild, moderate, and severe AD as reported by dermatologists AD: Atopic dermatitis

The survey was completed by 150 dermatologists, with 40 (26.67%) from the North, 45 (30.00%) from the South, 26 (17.33%) from the East, and 39 (26.00%) from the West of India (data available on request). The mean number of years of experience of the practicing dermatologists was 17.19±10.85 years. Among the 150 dermatologists surveyed, 57.33% reported that 5%-10% of their patients presented with AD, while 22.67% noted a prevalence between 10% and 15%. Pediatric-onset AD was reported to be the most common, with 44.67% of dermatologists indicating that more than 60% of their cases began in childhood. Adolescent-onset AD and adult-onset AD were less frequent and were reported to be less than 20% by 52.67% and 64.67% of dermatologists, respectively. In terms of severity, 34.67% of dermatologists indicated that 41-60% of their patients with AD had mild disease, while 46.67% reported that 21-40% of their patients had moderate AD. Severe AD was the least common, with 73.33% reporting that fewer than 20% of their patients fell into this category (Figure 1).

**Figure 2:**
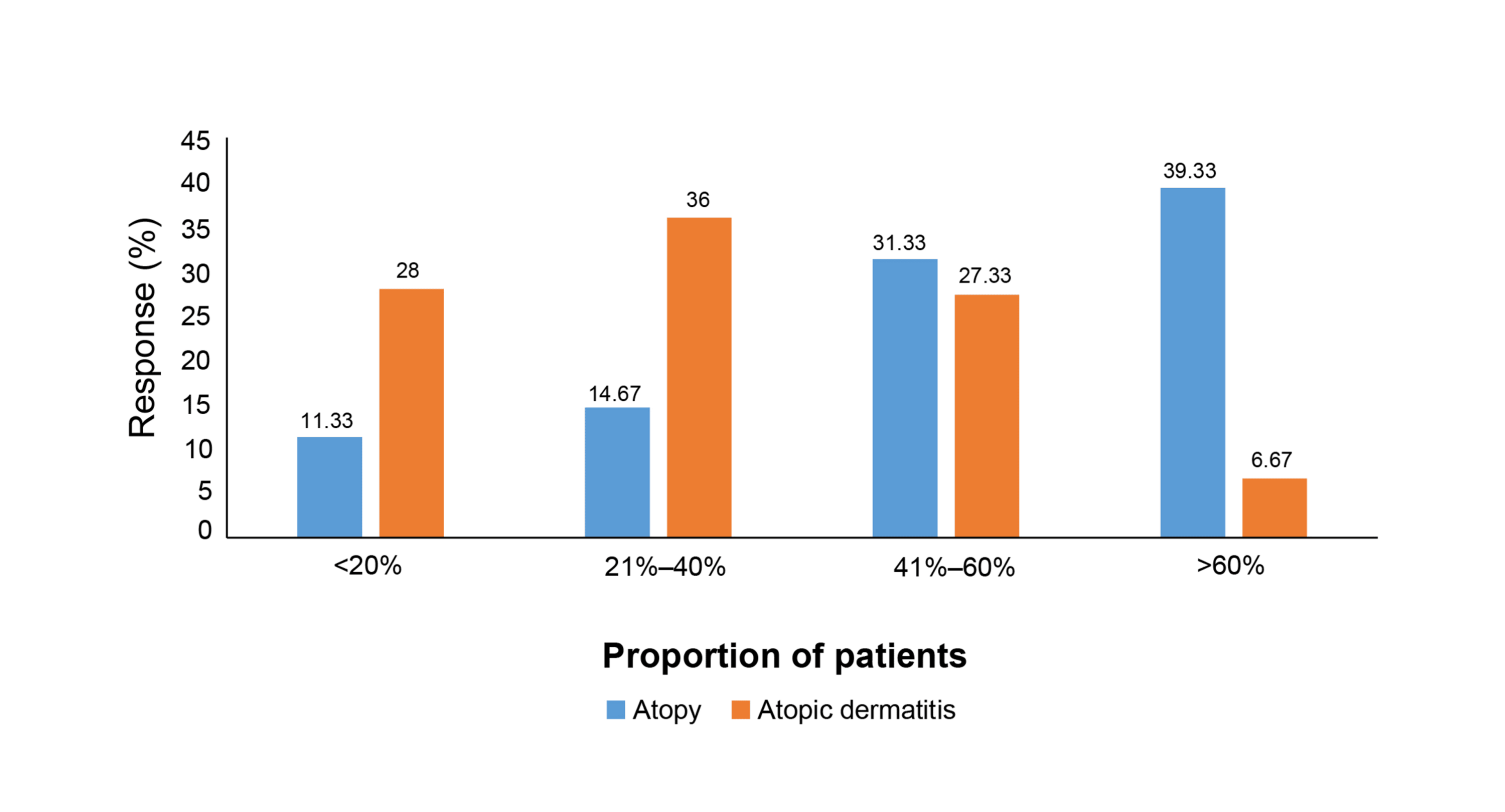
Proportion of patients having a family history of atopy or AD as reported by dermatologists AD: Atopic dermatitis.

Family history played a significant role, with 39.33% of dermatologists noting that over 60% of their patients had a family history of atopy, while 36% reported that 21-40% had a family history of AD (Figure 2). 

The responses to the questionnaire are outlined in Table 1.

**Table 1:** Questionnaire-epidemiology and demographics AD: Atopic dermatitis.

Sl. No.	Question		Options			
1	What percentage of the total patients at your clinical practice suffer from AD?	Categories	<5%	5–10%	10–15%	>15%
		Response, n (%)	19 (12.67)	86 (57.33)	34 (22.67)	11 (7.33)
2	Among all the patients with AD in your practice, how many have a pediatric onset, an adolescent onset, and an adult onset of the disease?	Categories	<20%	21–40%	41–60%	>60%
		Pediatric-onset, n (%)	13 (8.67)	27 (18.00)	38 (25.33)	67 (44.67)
		Adolescent-onset, n (%)	79 (52.67)	53 (35.33)	11 (7.33)	0 (0)
		Adult-onset, n (%)	97 (64.67)	38 (25.33)	7 (4.67)	2 (1.33)
3	What proportion of AD patients have mild, moderate, and severe disease?	Categories	<20%	21–40%	41–60%	>60%
		Mild, n (%)	16 (10.67)	32 (21.33)	52 (34.67)	47 (31.33)
		Moderate, n (%)	61 (40.67)	70 (46.67)	13 (8.67)	3 (2)
		Severe, n (%)	110 (73.33)	34 (22.67)	2 (1.33)	1 (0.67)
4	What proportion of patients have a family history of atopy or AD?	Categories	<20%	21–40%	41–60%	>60%
		Atopy, n (%)	17 (11.33)	22 (14.67)	47 (31.33)	59 (39.33)
		AD, n (%)	42 (28)	54 (36)	41 (27.33)	10 (6.67)

Clinical presentation and symptoms

**Figure 3:**
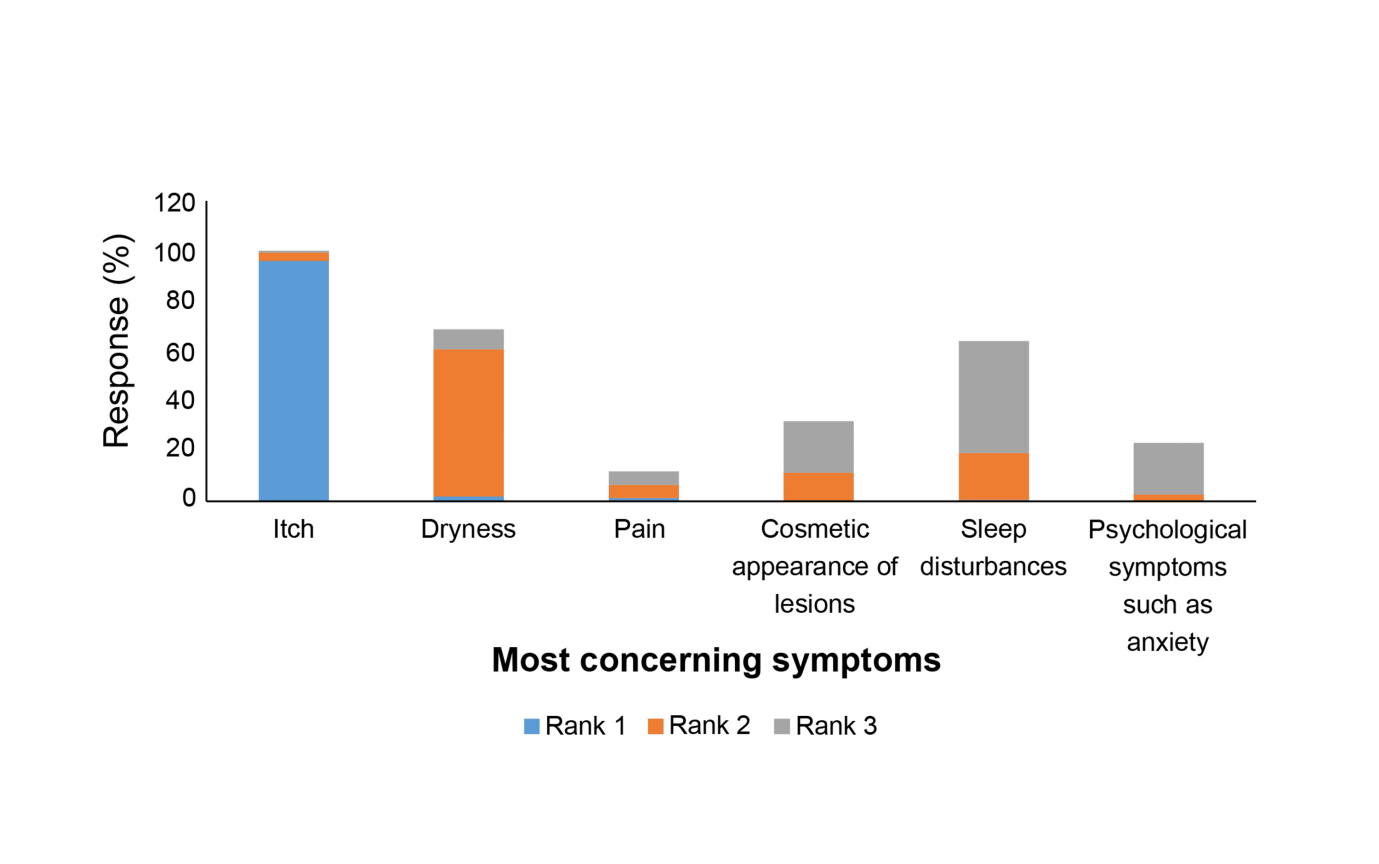
Top three most concerning signs and symptoms of AD as reported by dermatologists AD: Atopic dermatitis

The most common signs and symptoms of AD, as reported by dermatologists, were itch (96%), followed by dryness (58.67%) and sleep disturbances (44.67%) (Figure 3).

**Figure 4:**
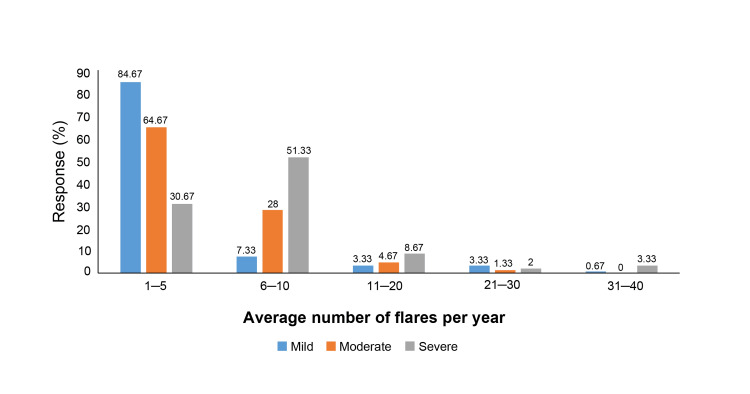
Average number of flare-ups per year noted in patients with AD as per disease severity AD: Atopic dermatitis.

A strong correlation between itch and disease severity was reported, with 70.67% of dermatologists reporting it in more than 60% of their patients. Regarding flare-up frequency, most mild AD cases experienced one to five flare-ups per year, as reported by 84.67% of dermatologists. Moderate cases had one to five flare-ups annually, according to 64.67% of the dermatologists, while severe cases showed six to 10 flare-ups per year, as per 51.33% of dermatologists (Figure 4).

Hot and humid weather was ranked first by 49.33% of dermatologists as the most influential factor that impacted AD flare-ups, followed by stress (34%) and a history of atopy (35.33%). Responses to the questionnaire are outlined in Table 2.

**Table 2:** Questionnaire-clinical presentation and symptoms AD: Atopic dermatitis.

Sl. No.	Questions		Options				
1	Choose the top three most concerning signs/symptoms of AD.	Categories	Rank 1	Rank 2	Rank 3		
		Itch, n (%)	144 (96.00)	5 (3.33)	1 (0.67)		
		Dryness, n (%)	3 (2.00)	88 (58.67)	12 (8.00)		
		Pain, n (%)	2 (1.33)	8 (5.33)	8 (5.33)		
		Cosmetic appearance of lesions, n (%)	0 (0.00)	17 (11.33)	31 (20.67)		
		Sleep disturbances, n (%)	1 (0.67)	28 (18.67)	67 (44.67)		
		Psychological symptoms such as anxiety, n (%)	0 (0.00)	4 (2.67)	31 (20.67)		
2	In what proportion of patients does the itch correlate with disease severity?	Categories	<20%	21%–40%	41%–60%	>60%	
		Response, n (%)	4 (2.67)	4 (2.67)	25 (16.67)	106 (70.67)	
3	What is the average number of flares/years noted in a patient with AD?	Categories	1–5	6–10	11–20	21–30	31–40
		Mild, n (%)	127 (84.67)	11 (7.33)	5 (3.33)	5 (3.33)	1 (0.67)
		Moderate, n (%)	97 (64.67)	42 (28.00)	7 (4.67)	2 (1.33)	0 (0.00)
		Severe, n (%)	46 (30.67)	77 (51.33)	13 (8.67)	3 (2.00)	5 (3.33)
4	Please rank the top three factors with an impact on flares of AD.	Categories	Rank 1	Rank 2	Rank 3		
		Hot and humid weather, n (%)	74 (49.33)	13 (8.67)	21 (14.00)		
		Stress, n (%)	37 (24.67)	51 (34.00)	32 (21.33)		
		Food allergens, n (%)	10 (6.67)	25 (16.67)	26 (17.33)		
		Pets, n (%)	5 (3.33)	8 (5.33)	11 (7.33)		
		Coexisting autoimmune conditions, n (%)	4 (2.67)	16 (10.67)	7 (4.67)		
		History of atopy, n (%)	20 (13.33)	37 (24.67)	53 (35.33)		

Healthcare utilization and challenges

Based on the survey results, most dermatologists reported that up to 20% of AD referrals were from general physicians, and comparatively fewer referrals came from pediatricians. Managing referred patients presented several challenges. The most common issues reported by dermatologists included delayed diagnosis (74.67%), incorrect treatment (74%), and a preference for alternative medicine (73.33%). Additionally, 64% of dermatologists mentioned undertreatment as a challenge, while 23% noted instances of overtreatment in referred patients. The responses to the questionnaire are outlined in Table 3.

**Table 3:** Questionnaire-Management of AD AD: Atopic dermatitis; DMARD: Disease-modifying antirheumatic drug; JAK: Janus kinase; PDE-4: Phosphodiesterase-4; TCI: Topical calcineurin inhibitor; TCS: Topical corticosteroid.

Sl. No.	Questions		Options				
1	What proportion of your AD patients are referred from other specialties?	Categories	<20%	21%–40%	41%–60%	>60%	
		Pediatrician, n (%)	79 (52.67)	45 (30.00)	18 (12.00)	2 (1.33)	
		General physician, n (%)	100 (66.67)	38 (25.33)	5 (3.33)	1 (0.67)	
2	What are the common challenges you face while managing referred patients?	Categories	Delayed diagnosis	Incorrect treatment	Under-treatment	Over-treatment	Preference for alternative medicine
		Response, n (%)	112 (74.67)	111 (74.00)	96 (64.00)	35 (23.00)	110 (73.33)
3	What percentage of patients have an existing medication history of corticosteroids for AD?	Categories	<20%	21%–40%	41%–60%	>60%	
		Topical, n (%)	5 (3.33)	7 (4.67)	21 (14)	108 (72)	
		Oral, n (%)	50 (33.33)	58 (38.67)	25 (16.67)	12 (8)	
4	What is the preferred first-line therapy for AD management in patients?	Categories	Mild (%)	Moderate (%)	Severe (%)		
		TCIs, n (%)	102 (68.00)	78 (52.00)	55 (36.67)		
		TCS, n (%)	122 (81.33)	113 (75.33)	89 (59.33)		
		Topical JAK inhibitors, n (%)	12 (8.00)	37 (24.67)	18 (12.00)		
		Oral JAK inhibitors, n (%)	1 (0.67)	26 (17.33)	97 (64.67)		
		Oral PDE-4 inhibitors, n (%)	13 (8.67)	48 (32.00)	47 (31.33)		
		DMARDs, n (%)	0 (0.00)	14 (9.33)	64 (42.67)		
		Other advanced therapies, n (%)	0 (0.00)	0 (0.00)	0 (0.00)		
5	What percentage of your patients with AD are lost to follow-up because of nonadherence to the treatment regimen?	Categories	<25%	25%–50%	50%–75%	>75%	
		Response, n (%)	72 (48)	66 (44)	11 (7.33)	1 (0.67)	
6	Enlist the proportion of patients with nonadherence to the treatment.	Categories	<20%	21%–40%	41%–60%	>60%	
		Steroid phobia, n (%)	61 (40.67)	53 (35.33)	19 (12.67)	4 (2.67)	
		Lack of awareness about the chronicity of disease, n (%)	41 (27.33)	61 (40.67)	28 (18.67)	13 (8.67)	
		Cost, n (%)	45 (30)	63 (42)	21 (14)	13 (8.67)	
		Others	Preference for alternative medicine: 25.37%				
			Cost and accessibility: 6.67%				
			Chronicity and nature of disease: 7.33%				
			Treatment-related issues: 7.33%				
			Counseling and support: 3.33%				
7	Do you think a newer steroid-sparing option would improve patient adherence?	Categories	Yes	No	Maybe		
		Response, n (%)	117 (78.00)	2 (1.33)	31 (20.67)		

Management and adherence

**Figure 5:**
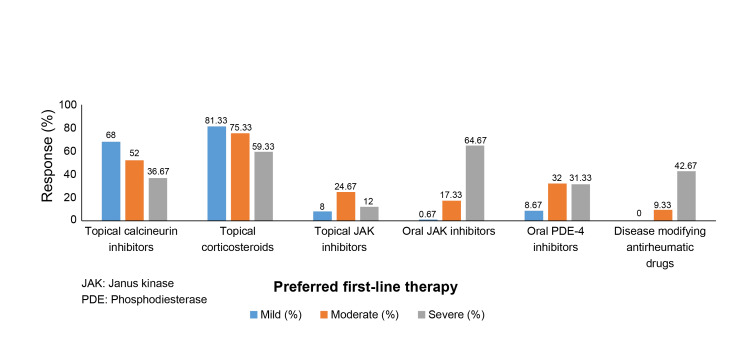
Preferred first-line therapy for AD management as per disease severity in patients as reported by dermatologists AD: Atopic dermatitis; JAK: Janus kinase; PDE-4: Phosphodiesterase-4.

Dermatologists (72%) reported that more than 60% of their patients with AD had a history of using topical corticosteroids (TCS). In comparison, 38.67% indicated that 21-40% of their patients had used oral corticosteroids. As the first-line therapy, TCS was the preferred option for mild AD by 81.33% of dermatologists and for moderate AD by 75.33%. Topical calcineurin inhibitors (TCIs) were also commonly used by 68% and 52% of dermatologists for mild and moderate AD, respectively. Meanwhile, oral Janus kinase (JAK) inhibitors were favored for severe AD by 64.67% of dermatologists (Figure 5).

**Figure 6:**
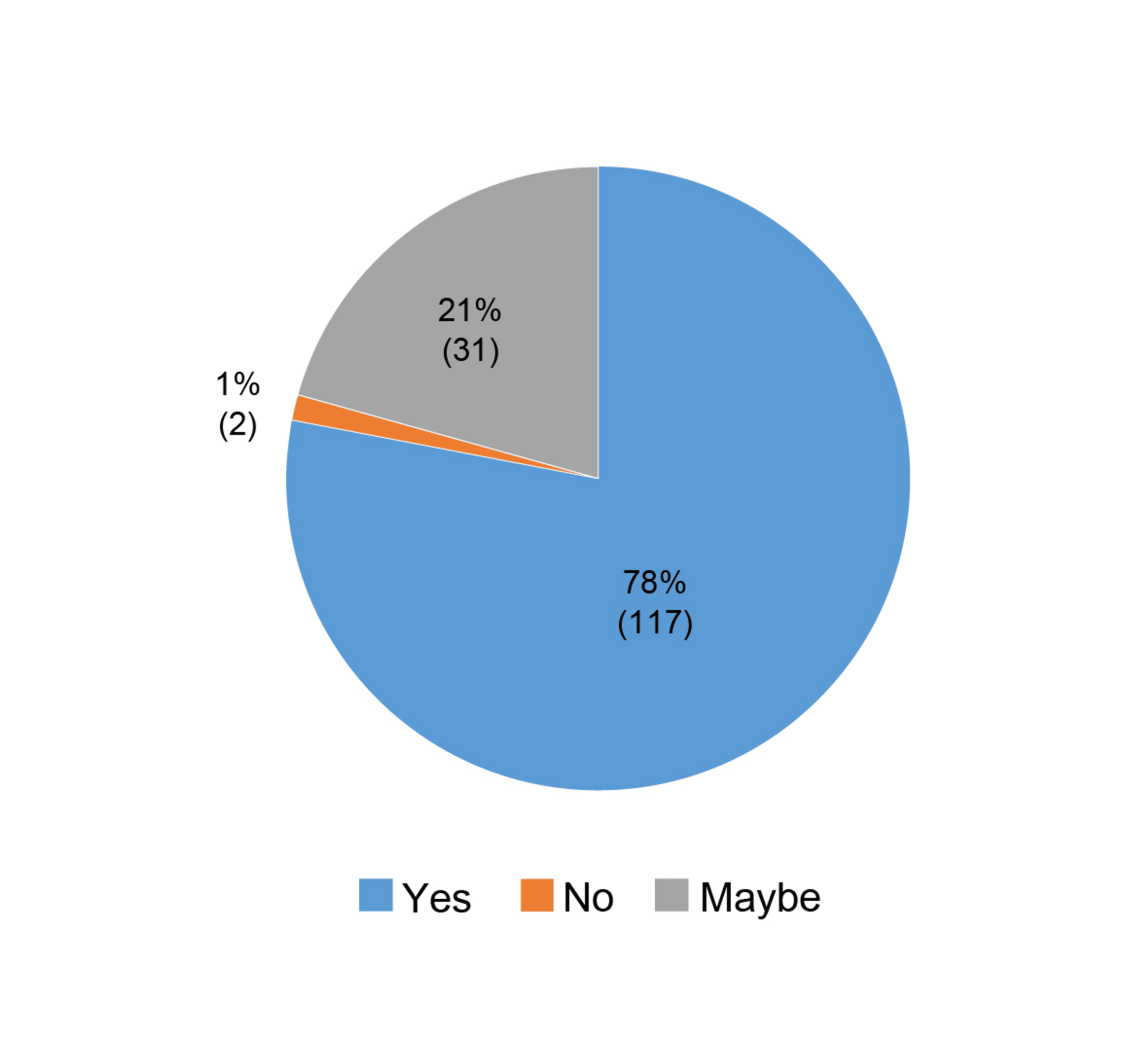
Need for newer steroid-sparing options as expressed by dermatologists (%).

Dermatologists predominantly recommended TCIs and moisturizers as maintenance therapies to control the disease. Nonadherence to treatment was noted as a significant challenge, with 48% of dermatologists reporting that up to 25% of their patients were lost to follow-up due to nonadherence. The lack of awareness of AD chronicity and cost barriers were the common reasons for nonadherence observed among 21-40% of patients. Steroid phobia was also reported in up to 20% of patients (TCS phobia or steroid phobia refers to the negative feelings and beliefs related to TCSs experienced by patients and patients' caregivers). A preference for alternative medicine was among the other most commonly reported reasons. Finally, 78% of dermatologists believed that newer steroid-sparing options would improve patient adherence, signaling a demand for alternatives in managing AD (Figure 6).

The responses to the questionnaire are outlined in Table 3.

Variations across different zones (North, South, East, and West)

The analysis of responses from dermatologists in the North, South, East, and West zones of India revealed regional variations in the onset and management of AD. The prevalence of pediatric-onset AD was high across all zones. In the South zone, 48.9% of dermatologists stated that 21-40% of their patients presented with adolescent-onset AD. Similarly, 46.7% of dermatologists stated that 21-40% of patients reported adult-onset AD. Compared with other regions, the incidences of adolescent- and adult-onset AD were more frequent in the South zone. Nonadherence to treatment, particularly due to steroid phobia, was also higher in the South zone. In terms of treatment preferences (Table 4), oral phosphodiesterase-4 (PDE-4) inhibitors were more commonly used in the South zone as a first-line treatment for mild AD (28.9%) than in the other zones, where TCS and TCIs remained the primary choices. For moderate AD, the use of topical JAK inhibitors (42.5%) and oral PDE-4 inhibitors (50.0%) was notably higher in the North zone. In contrast, dermatologists preferred oral JAK inhibitors more in the East zone (33.3%) than in the other zones. Conversely, the East zone reported the lowest preference for oral PDE-4 inhibitors (3.9%) in moderate AD cases. For severe AD, the preference for oral PDE-4 inhibitors was highest in the North zone (62.5%). Oral JAK inhibitors were less favored in the South zone, where disease-modifying antirheumatic drugs (DMARDs) were more commonly chosen (60.0%). This contrasted with other zones, where oral JAK inhibitors were more frequently used.

**Table 4:** Preferred first-line therapy for patients with AD across different zones AD: Atopic dermatitis; DMARD: Disease-modifying antirheumatic drug; JAK: Janus kinase; PDE-4: Phosphodiesterase-4; TCI: Topical calcineurin inhibitor; TCS: Topical corticosteroid.

	Mild				Moderate				Severe			
	North	East	South	West	North	East	South	West	North	East	South	West
TCIs, n (%)	29 (72.5)	19 (73.1)	37 (82.2)	17 (43.6)	14 (35.0)	14 (53.9)	31 (68.9)	19 (48.7)	9 (22.5)	11 (42.3)	22 (48.9)	14 (35.9)
TCS, n (%)	31 (77.5)	19 (73.1)	38 (84.4)	35 (89.7)	19 (47.5)	21 (80.8)	38 (84.4)	35 (89.7)	11 (27.5)	15 (57.7)	35 (77.8)	28 (71.8)
Topical JAK inhibitors, n (%)	0 (0.0)	3 (11.5)	2 (4.4)	7 (17.9)	17 (42.5)	2 (7.7)	5 (11.1)	13 (33.3)	6 (15.0)	3 (11.5)	2 (4.4)	7 (17.9)
Oral JAK inhibitors, n (%)	0 (0.0)	0 (0.0)	1 (2.2)	0 (0.0)	3 (7.5)	8 (30.8)	6 (13.3)	9 (23.1)	30 (75.0)	17 (65.4)	19 (42.2)	31 (79.5)
Oral PDE-4 inhibitors, n (%)	0 (0.0)	0 (0.0)	13 (28.9)	0 (0.0)	20 (50.0)	1 (3.9)	18 (40.0)	9 (23.1)	25 (62.5)	5 (19.2)	9 (20.0)	8 (20.5)
DMARDs, n (%)	0 (0.0)	0 (0.0)	0 (0.0)	0 (0.0)	3 (7.5)	4 (15.4)	4 (8.9)	3 (7.7)	17 (42.5)	8 (30.8)	27 (60.0)	12 (30.8)

## Discussion

AD is a chronic and heterogeneous skin condition influenced by environmental and genetic factors, leading to varying clinical symptoms across patients [1,11]. This study aimed to assess the perspectives of Indian dermatologists on the burden, clinical presentation, and management of AD. While these results provide important insights, they should be interpreted in the context of the study’s cross-sectional, self-reported design, which limits causal inference and may introduce recall bias. Nevertheless, the findings can contribute to a preliminary understanding of the current scenario in India. For the purpose of this survey, “burden” was defined as the overall clinical impact of AD on the patient’s daily functioning, quality of life, and treatment needs, as perceived by the dermatologist. “Flare-up” was defined as a period of acute worsening of AD symptoms, such as increased itch, erythema, and lesion severity, that typically required adjustment or escalation of therapy.

It was observed in this study that the disease primarily manifests in early childhood. Approximately 80% of patients experience symptom onset within the first few years of life [1]. Many of these patients may experience remission during adolescence (about 60%). However, the incidence of adult-onset AD is reported to be increasing [12]. This study found regional variations in the onset and management of AD across India, with the South zone reporting a higher prevalence of adolescent- and adult-onset AD compared with other zones. Our findings of regional variation, particularly higher adolescent- and adult-onset AD in the South zone, are noteworthy but should be viewed cautiously, as they are based on physician recall rather than patient-level registry or longitudinal data. The observed regional variations may still highlight the importance of considering individual environmental exposures and genetic predispositions when managing the disease. This aligns with findings across the geographically diverse United States, where climatic conditions have been reported to be a predictor of AD office visits, similar to what Indian dermatologists reported in this survey [13]. Consistent with this study, family history of atopy, including maternal and paternal history, has been strongly associated with an increased risk of developing AD [14].

One of the critical signs/symptoms impacting patients with AD is pruritus, which significantly affects the quality of life by causing sleep disturbances [15]. This relationship between AD and sleep disorders may be bidirectional, forming a vicious cycle where the lack of sleep exacerbates symptoms and increases itchiness [16]. The results of this study reflect similar trends, with dermatologists highlighting itch as the most concerning symptom, followed by dryness and sleep disturbances. These symptoms have been reported to be most burdensome and can significantly impair the overall quality of life of patients with AD [17]. Such symptom patterns align with prior Indian and international studies that underscore the centrality of itch in disease burden, although our study cannot quantify its exact impact on quality of life due to the absence of patient-reported outcome measures. Comparable findings were also observed in an international study on patient-reported burden, where itch, dryness, and sleep disturbance were the top-ranked symptoms [18]. Similar to sleep disturbance, AD also has a bidirectional psychological impact, with the skin condition negatively affecting mental health, which in turn can exacerbate flare-ups [19]. This necessitates the need for adequate treatment and symptom relief to break the vicious cycle of sleep disturbance/stress and flare-ups. Both endogenous and environmental factors can trigger or exacerbate AD flare-ups [20]. Factors such as hot and humid weather, as well as psychological stress, were reported by dermatologists across different zones of India as common triggers for flare-ups. These triggers exacerbate pruritus and skin inflammation, which further complicates disease management [20]. While this finding is consistent with earlier Indian studies, our survey did not measure objective environmental exposures, so these associations remain based on clinical impressions rather than measured data.

A considerable challenge highlighted in the responses from the surveyed dermatologists was the delayed diagnosis and referral of AD, reported by 74.67% of respondents as a common issue. This delay is often compounded by patients' misconceptions and a lack of awareness about the condition, consistent with findings from both global and Indian literature [5,6,21,22]. Many patients visit multiple doctors before receiving a confirmed diagnosis, often leading to inappropriate treatments that worsen their condition [6]. General practitioners who receive limited dermatological training may contribute to delays in diagnosis and treatment by failing to refer patients to dermatologists on time [23]. In addition, patients frequently turn to alternative medicine and discontinue their prescribed medications, further complicating their disease management [6]. Our findings reinforce these documented barriers, but cannot establish their prevalence at a national level, given the convenience sampling approach.

In terms of treatment, TCS remains the cornerstone of AD management, especially during acute flare-ups [2]. TCS suppresses the inflammatory immune response and provides rapid symptom relief for most patients. Long-term studies have shown that proactive treatment with TCS can effectively prevent flare-ups [24]. TCIs, such as pimecrolimus and tacrolimus for patients with moderate-to-severe AD, have proven effective in both short-term and long-term treatments [25]. Despite the efficacy of these topical treatments, managing moderate-to-severe AD often requires systemic therapies, such as immunosuppressive treatments, which are associated with significant adverse effects [26]. Newer treatments, such as those with JAK inhibitors, have emerged as promising therapeutic options, particularly for patients who do not respond to traditional systemic therapies or biologics [26]. Oral JAK inhibitors, including abrocitinib and upadacitinib, have shown significant efficacy in treating AD [26]. However, as our study was not designed to evaluate treatment effectiveness, these therapy-related observations reflect dermatologist preferences and perceptions rather than comparative clinical outcomes.

However, poor adherence to treatment remains a major barrier to achieving favorable outcomes in AD management [27]. Factors contributing to nonadherence include concerns about side effects, treatment costs, and a lack of understanding of the chronic nature of the disease [28]. In this study, steroid phobia and the use of alternative medicines were cited as strong reasons for nonadherence, particularly in the South zone. Studies have shown that adherence to topical treatment drops considerably within the first few days of beginning treatment, further emphasizing the need for patient education and support [28]. This is consistent with findings, where steroid phobia has been reported in up to 83.7% of patients who express fears about corticosteroid use, contributing to underuse and treatment failure [29]. Given the survey-based nature of our data, the adherence estimates should be interpreted as indicative trends rather than definitive prevalence figures.

Beyond traditional corticosteroids, steroid-sparing alternatives offer improved treatment adherence for patients with AD. For mild-to-moderate AD, crisaborole 2% ointment, a PDE-4 inhibitor approved by the Food and Drug Administration, has demonstrated promising results in the Indian population. A prospective, open-label study conducted at a tertiary care center in Eastern India showed statistically significant improvement in SCORing Atopic Dermatitis (SCORAD), Investigator's Static Global Assessment (ISGA), and Eczema Area and Severity Index (EASI) scores after 28 days of twice-daily crisaborole (2%) application, although complete resolution was achieved in only 20% of patients [30]. For moderate-to-severe AD, biologics and JAK inhibitors offer better disease management. Dupilumab, the first targeted biologic therapy approved for AD, has shown significant efficacy in Indian patients with moderate-to-severe AD across multiple centers in Kolkata and Bangalore, with 68% of patients achieving EASI-75 after six months of treatment and substantial improvement in quality of life. These patients had previously failed to respond adequately to at least one systemic immunomodulator [31]. More recently, JAK inhibitors like abrocitinib have emerged as alternatives to biologics, with global data suggesting faster itch relief compared to dupilumab in patients who did not show improvement with medicated creams or ointments [32]. Treatment-emergent adverse events associated with JAK inhibitors have been reported mainly to be mild to moderate and include acne, nausea, headache, upper respiratory tract infection, and, to a lesser degree, herpes infection and selected laboratory abnormalities [33]. In terms of the availability of these molecules in India, dupilumab was approved in 2023 for the treatment of moderate-to-severe AD in adults whose disease is not adequately controlled with topical prescription therapies [34]. Crisaborole has been recently approved in India [35]. Similarly, abrocitinib has recently received marketing authorization in India [36]. Such advancements are crucial for improving treatment outcomes, especially for patients with severe diseases. While these therapeutic advances are promising, our study cannot evaluate their comparative effectiveness or safety, and future head-to-head clinical studies in Indian populations will be critical to guide practice.

Effective AD management requires a comprehensive understanding of individual patients’ risk factors, environmental triggers, and adherence challenges. It is critical to implement a stepwise treatment approach, starting with acute management and transitioning to proactive maintenance therapy to prevent relapse [25]. Ongoing research into more effective and safer long-term treatment options, including newer steroid-sparing agents, is essential for improving the quality of life of patients with moderate-to-severe AD [26]. Future research should aim to validate these findings through larger, probability-sampled, multi-center studies incorporating both patient-reported and objective clinical outcomes to provide a more definitive national perspective.

This study has some limitations that should be acknowledged. First, the cross-sectional survey design may be subject to recall bias as it relies on self-reported data from dermatologists, which may not always accurately reflect real-world clinical practice. The absence of direct clinical verification of reported prevalence, severity, and treatment patterns further limits the robustness of the findings. Additionally, the survey was limited to a specific number of dermatologists and lacked a sample size calculation, which may not fully represent the diversity of experiences and management strategies across India. The use of the convenience sampling method and voluntary participation could introduce selection bias, as dermatologists with a specific interest in AD may have been more likely to respond. Geographic variations in terms of access to healthcare facilities and socioeconomic factors, which could influence the diagnosis and treatment of AD, were not thoroughly addressed. Finally, the study does not account for patient perspectives, which are crucial for understanding the full impact of AD and the barriers to effective management. These limitations may limit the generalizability of the findings to other settings or the overall national pattern and should receive due consideration while interpreting the results from this study. Future studies incorporating both healthcare professionals’ and patients’ viewpoints with longitudinal data, random sampling, and an externally validated survey instrument would provide a more comprehensive understanding of AD management in India.

## Conclusions

The findings of this survey highlight expert perceptions of unmet needs in the management of AD across India. A substantial burden of pediatric-onset AD was reported, with most cases falling within the mild-to-moderate severity range. Managing referred patients presents challenges, particularly in cases of delayed diagnosis and treatment, which can exacerbate the condition. Nonadherence to treatment, influenced by factors such as steroid phobia and misconceptions about the disease, further complicates the management of AD. There is a clear need for effective maintenance therapies that ensure better long-term disease control. Introducing targeted therapies, such as JAK inhibitors and newer steroid-sparing options, could potentially enhance patient outcomes and address the challenges faced by both patients and healthcare providers. Improved education and awareness, alongside the availability of these advanced therapies, are essential to optimizing the management of AD and improving the quality of life of patients with AD in India.

## Appendices

**Table 5:** Atopic dermatitis questionnaire

Sl. No.	Question	Options	Response
1	Details of the doctor:	Years of Experience	
		Hospital/Clinic	
2	What percentage of total patients at your clinical practice suffer from Atopic Dermatitis?	<5%	
		5-10%	
		10-15%	
		>15%	
3	Amongst all the patients with Atopic Dermatitis in your practice, how many have a pediatric-onset, adolescent onset and adult-onset of the disease:	Pediatric-onset	
		Adolescent-onset	
		Adult-onset	
4	What proportion of AD patients are mild, moderate and severe?	Mild	
		Moderate	
		Severe	
5	What proportion of patients have a Family history of atopy or AD:	Family history of atopy (%)	
		Family history of AD (%)	
6	Choose the top 3 most concerning symptoms of Atopic Dermatitis	Itch	
		Dryness	
		Pain	
		Cosmetic appearance of lesions	
		Sleep disturbances	
		Psychological symptoms such as Anxiety	
7	In what proportion of patients does the itch correlate with disease severity?		
8	What are the average number of flares noted in a patient with?	Mild	
		Moderate	
		Severe	
9	Please rank the top 3 factors with an impact on flares of Atopic Dermatitis	Hot & Humid	
		Stress	
		Food allergens	
		Pets	
		Coexisting autoimmune	
		History of atopy	
10	What % of your AD patients are referred from other specialties?	Pediatricians	
		General physicians	
		Not applicable	
11	Which are the common challenges that you face whiles managing referred patients? (Please select all possible options)	Delayed diagnosis	
		Incorrect treatment	
		Under treatment	
		Over Treatment	
		Preference for alternative medicine	
12	What % of patients have an existing medication history of corticosteroids for AD?	Topical	
		Oral	
13	What is Preferred first line therapy for AD management in patients?	Mild	TCIs
			TCS
			Topical JAK
			Oral JAKi
			Oral PDE-4
			DMARDs
			Other advanced therapies (please specify)
		Moderate	TCIs
			TCS
			Topical JAK
			Oral JAKi
			Oral PDE-4
			DMARDs
			Other advanced therapies (please specify)
		Severe	TCIs
			TCS
			Topical JAK
			Oral JAKi
			Oral PDE-4
			DMARDs
			Other advanced therapies (please specify)
14	What is your Preferred choice of maintenance therapy once the flare is under control?	Drug	
		Duration	
15	What percentage of your patients with Atopic Dermatitis are lost to follow up because of non-adherence to the treatment regime?	<25 %	
		25-50 %	
		50-75 %	
		>75 %	
16	Enlist the proportion of patients with non-adherence to the treatment?	Steroid Phobia (%)	
		Lack of Pt awareness on the chronicity of the disease (%)	
		Cost of the therapy	
		Others- please specify	
17	Do you think a newer steroid sparing option would improve the patient adherence?	Yes	
		No	
